# The metabolic profiles and body composition of non-obese metabolic associated fatty liver disease

**DOI:** 10.3389/fendo.2024.1322563

**Published:** 2024-02-05

**Authors:** Yujuan Zhang, Liulan Xiang, Fang Qi, Yutian Cao, Wenhui Zhang, Tiansu Lv, Xiqiao Zhou

**Affiliations:** ^1^ Department of Endocrinology, Jiangsu Province Hospital of Chinese Medicine, Affiliated Hospital of Nanjing University of Chinese Medicine, Nanjing, China; ^2^ The First Clinical Medical College of Nanjing University of Chinese Medicine, Nanjing, China

**Keywords:** MAFLD, non-obese, body composition, metabolic profiles, hepatic fibrosis

## Abstract

**Background/purpose:**

Metabolic-associated fatty liver disease (MAFLD) is a major cause of chronic liver disease worldwide and is generally thought to be closely related to obesity and diabetes. However, it also affects non-obese individuals, particularly in Asian cultures.

**Methods:**

Healthy physical examination subjects and MAFLD patients were included in the endocrinology department of Jiangsu Provincial Hospital of Traditional Chinese Medicine. MAFLD was defined as fatty liver in imaging without virus infection, drug, alcohol, or other known causes of chronic liver disease. Non-obese MAFLD was defined as MAFLD in non-obese subjects (BMI<25 kg/m2).

**Results:**

The final analysis comprised 1047 participants in total. Of 946 MAFLD patients, 162 (17.12%) were diagnosed with non-obese MAFLD. Non-obese MAFLD patients were older, had lower alanine aminotransferase (ALT), triglyceride, and waist circumference, but had higher high density lipoprotein cholesterol (HDL-c) than obese MAFLD patients. Compared with non-obese healthy controls, non-obese MAFLD patients had higher BMI, ALT, gamma-glutamyl transferase (GGT), uric acid (UA), triglycerides (TG), and low density lipoprotein cholesterol (LDL-c). In terms of body composition, body fat mass (BFM), waist-hip ratio (WHR), percent body fat (PBF), visceral fat area (VFA), and fat mass index (FMI) were lower in non-obese healthy controls than non-obese MAFLD patients. A binary logistic regression analysis revealed that non-obese MAFLD was linked with lower GGT and higher HDL-c.

**Conclusion:**

In this study cohort, non-obese MAFLD was present at a prevalence of 13.90%. In contrast to non-obese healthy controls, non-obese MAFLD patients exhibited different metabolic profiles, but they also had different body compositions.

## Introduction

Metabolic associated fatty liver disease (MAFLD), formerly known as non-alcoholic fatty liver disease (NAFLD), is a major cause of chronic liver disease worldwide. The latest epidemiological studies show that MAFLD affects more than 1/3 of the world’s population and is associated with the development of many types of cancer. Previous studies have generally suggested that the development of MAFLD is strongly associated with obesity, which is commonly measured using body mass index (BMI). However, a growing body of research suggests that MAFLD can also occur in non-obese people, especially in the Asian population. Around 8%-19% of Asians have non-obese MAFLD (1). Although non-obese MAFLD patients are generally considered to have a milder metabolic profile than obese patients, it is still possible to progress to hepatitis, liver fibrosis, and even liver cancer.

Transient elastography (Fibroscan, TE) with controlled attenuation parameter (CAP) has demonstrated good accuracy in quantifying the levels of liver steatosis and fibrosis in patients with MAFLD (2, 3). The assessment of body composition is an important reference for an individual’s health, nutritional status, and physical fitness (4). The relationship between non-obese MAFLD and body composition has rarely been studied. The InBody 770 is a universal, convenient, and highly accurate bioelectrical impedance analyzer that is currently a common method for measuring body composition (5).

Therefore, we aim to explore the metabolic characteristics and body composition of non-obese individuals with MAFLD compared with obese MAFLD patients and non-obese healthy controls.

## Method

### Patients and study design

**Figure 1:**
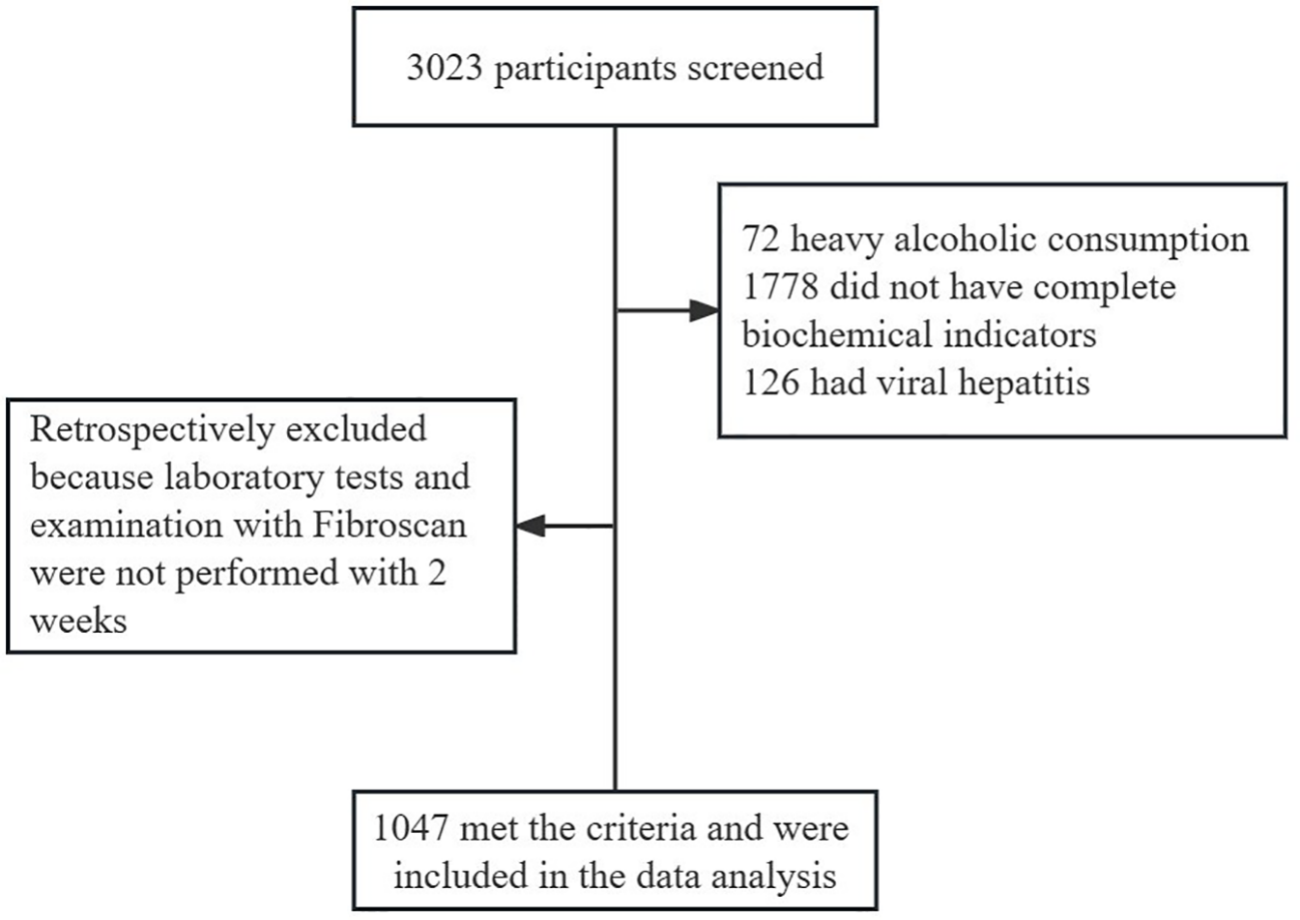
Patient selection flowchart.

This was a cross-sectional study. We ultimately included 1047 participants in this review. The subject screening flowchart is shown in **Figure 1**. Participants were recruited from patients treated in the Department of Endocrinology of Jiangsu Provincial Hospital of Traditional Chinese Medicine between June 2022 and February 2023, of whom 3023 underwent Fibroscan and InBody examinations. The participants included patients with MAFLD and healthy controls. The diagnosis of MAFLD refers to the 2020 edition of the International Expert Consensus Statement (6). For the present study, we only included participants with serologic results within two weeks of having Fibroscan and InBody tests. Patients with alcoholic fatty liver disease, viral hepatitis, autoimmune liver disease, liver tumors, and other chronic liver diseases leading to impaired liver function were excluded. Patients taking drugs associated with secondary MAFLD, such as corticosteroids, estrogens, amiodarone, and methotrexate, should be excluded. Participants with a weekly alcohol consumption of >210g for men and >140g for women were excluded. Subjects who did not have complete data on serological indicators were also excluded. Venous blood was collected on an empty stomach for at least 8 hours. FIB-4, FAST, and LSM were used to determine the stage of liver fibrosis. The MAFLD participants were divided into two groups according to BMI (<25 kg/m^2^ non-obese, ≥25 kg/m^2^ obese) and were further compared with the non-obese healthy group.

This study complies with the ethical guidelines of the Declaration of Helsinki of 1975. The study was approved by the Ethics Committee of Jiangsu Provincial Hospital of Traditional Chinese Medicine.

### Laboratory testing

The serum biochemical indexes were measured by the automatic biochemical analyzer of the Laboratory Department of Jiangsu Provincial Hospital of Traditional Chinese Medicine. Platelets and hemoglobin were detected by automatic blood cell analyzer. Insulin resistance index (HOMA-IR) of ≤ 1 is considered normal, while an index > 2.0 can be interpreted as evidence of insulin resistance (7). HOMA-IR was calculated according to the formula: fasting insulin (μU/ml)×fasting glucose (mmol/L)/22.5. The FIB-4 index (8) and FAST score (9) were calculated using the following formulas according to previous reports. For FIB-4, cutoffs of<1.30 and *≥*2.67 were used as the rule-out and rule-in criteria for elevated liver stiffness (10). For FAST, we used ≤0.35 and ≥0.67 (11).


$$\text{FIB-}4=\text{Age}\times \text{AST}/\left[\text{PLT}\times {\left(\text{ALT}\right)}^{1/2}\right]$$

$$\text{FAST}=\frac{e^{-1.65+1.07\times \ln\left(LSM\right)+2.66*10^{-8}\times CAP^{3}-63.3\times AST^{-1}}}{1+e^{-1.65+1.07\times \ln\left(LSM\right)+2.66*10^{-8}\times CAP^{3}-63.3\times AST^{-1}}}$$

### Transient elastography

Fibroscan (Echosens, Paris, France) was used to determine Liver stiffness measurement (LSM) and controlled attenuation parameter (CAP), with a 3.5-MHz M probe for subjects with BMI<30 and a 2.5-MHz XL probe for those with BMI≥30. The subject was placed in the supine position, and the right hand was raised above the head to fully expose the liver area. The 7^th^ to 9^th^ intercostal areas of the anterior and midaxillary lines were selected for measurement. Results were considered reliable when at least 10 valid LSM and CAP values were measured and the interquartile range or median was less than 30%. Median CAP values at least 238, 259, and 292 dB/m were considered indicative of S1, S2, and S3 steatosis. A median LSM at least 7.3 kPa was considered indicative of significant (≥F2) fibrosis, whereas values at least 12.4 kPa and 17.5 kPa were considered indicative of F3 (advanced fibrosis) and F4 (cirrhosis).

### Body composition measurement

Height and body weight were measured with the participants wearing light clothing and no footwear. BMI was calculated by dividing the body weight in kilograms by the height in square meters (kg/m^2^). Body composition was determined by InBody 770 under constant conditions (proper hydration and the same time of day). Each participant was required to stand on the device with bare feet and hold the electrodes in their hands. The impedance data included body fat mass (BFM), fat free mass (FFM), visceral fat area (VFA), percent body fat (PBF), and skeletal muscle index (SMI).

### Statistical analyses

SPSS26.0 (SPSS Inc, Chicago, IL) was used for statistical analysis. Measurement data were expressed as mean value and t-test was used. The qualitative data were represented by percentage and the χ^2^ test was used. *P*<0.05 was considered statistically significant. The propensity score matching (PSM) method was used in this study to balance the baseline differences in a 1:2 ratio. And using binary logistic regression to explore the related factors of non-obese MAFLD.

## Results

Between January 2022 and February 2023, 3023 patients visited the Department of Endocrinology of Jiangsu Provincial Hospital of Traditional Chinese Medicine and completed the Fibroscan examination. There were 2287 MAFLD patients. Of them, 318 participants were categorized as non-obese MAFLD patients. 1341 patients without available data on liver function, lipid, FIB-4, or FAST score were excluded. A total of 1047 subjects were included in the final analysis. Our study had adequate sample size and statistical power to handle the factors of non-obese MAFLD.

1. Comparison of clinical characteristics and metabolic profiles between non-obese MAFLD and obese MAFLD patients

Compared with obese MAFLD patients, non-obese MAFLD patients were older and had higher high density lipoprotein (HDL), but lower waist circumference, hemoglobin, blood platelets, aspartate (AST), alanine aminotransferase (ALT), gamma-glutamyl transferase (GGT), uric acid (UA), triglycerides (TG), cholesterol (CHO), and glucose level. Compared with obese MAFLD patients, LSM, FIB-4 levels, and FAST score were also lower in non-obese MAFLD patients. The body composition indexes of non-obese MAFLD subjects were significantly lower than those of obese MAFLD subjects (**Table 1**).

**Table 1:** Comparison of clinical characteristics and body composition between non-obese MAFLD and obese MAFLD patients.

Characteristic	Unmatching			Matching		
	Non-obese MAFLD (n = 162)	Obese MAFLD (n = 784)	*P* value	Non-obese MAFLD (n = 95)	Obese MAFLD (n = 157)	*P* value
Demography						
Age, year	50.45 ± 12.33	38.38 ± 13.08	<0.001	58.81 ± 7.13	58.52 ± 6.73	0.742
Male, *n* (%)	70 (43.2%)	366 (46.7%)	0.419	35 (36.84%)	75 (47.77%)	0.090
Waist, cm	83.44 ± 4.91	102.12 ± 11.21	<0.001	83.39 ± 4.95	95.66 ± 8.36	<0.001
BMI	23.32 ± 1.32	30.61 ± 3.97	<0.001	23.21 ± 1.22	28.33 ± 2.98	<0.001
DM, *n* (%)	113 (69.8%)	343 (43.8%)	<0.001	75 (78.95%)	119 (75.79%)	0.439
HTN, *n* (%)	40 (24.7%)	186 (23.7%)	0.793	33 (34.74%)	73 (46.49%)	0.067
Cardiovascular and cerebrovascular diseases, *n* (%)	5 (3.1%)	19 (2.4%)	0.625	5 (5.26%)	18 (11.46%)	0.098
Laboratory						
Hemoglobin, g/L	141.49 ± 17.58	144.84 ± 16.02	0.018	139.62 ± 13.93	142.97 ± 13.76	0.063
Platelets, 10^9/L	232.61 ± 66.93	261.05 ± 69.22	<0.001	224.6 ± 61.50	213.87 ± 57.49	0.163
AST, U/L	23.79 ± 15.971	30.005 ± 23.09	0.001	22.17 ± 10.59	24.54 ± 16.35	0.208
ALT, U/L	28.82 ± 23.67	47.05 ± 44.11	<0.001	24.68 ± 15.17	31.99 ± 29.42	0.026
ALP, U/L	83.98 ± 22.52	87.22 ± 33.67	0.241	85.00 ± 23.20	87.89 ± 23.91	0.348
GGT, U/L	41.60 ± 40.76	50.47 ± 42.74	0.015	39.74 ± 32.49	43.71 ± 38.64	0.047
UA, μmol/L	323.78 ± 74.81	385.18 ± 103.68	<0.001	313.45 ± 76.57	330.92 ± 76.07	0.079
Triglycerides, mmol/L	1.92 ± 1.46	2.24 ± 1.95	0.048	1.85 ± 1.22	2.10 ± 1.65	0.194
CHO, mmol/L	5.01 ± 1.14	5.06 ± 0.97	0.533	4.99 ± 1.23	5.13 ± 1.02	0.338
HDL-c, mmol/L	1.34 ± 0.31	1.24 ± 0.26	<0.001	1.37 ± 0.32	1.28 ± 0.26	0.016
LDL-c, mmol/L	2.88 ± 0.79	3.02 ± 0.74	0.023	2.86 ± 0.86	2.98 ± 0.79	0.261
Glucose, mmol/L	7.00 ± 2.40	6.35 ± 2.14	0.001	7.27 ± 2.39	7.01 ± 2.14	0.372
CAP, dB/m	280.30 ± 34.72	308.48 ± 38.30	<0.001	277.68 ± 33.43	295.83 ± 34.86	<0.001
LSM, kPa	5.39 ± 1.78	7.06 ± 3.02	<0.001	5.37 ± 1.81	7.21 ± 3.37	<0.001
FIB-4	1.08 ± 0.54	0.74 ± 0.51	<0.001	1.29 ± 0.53	1.32 ± 0.61	0.686
FAST	0.13 ± 0.14	0.30 ± 0.26	<0.001	0.11 ± 0.13	0.20 ± 0.21	0.001
Body composition						
BFM	18.57 ± 3.59	31.68 ± 8.84	<0.001	18.82 ± 3.13	26.58 ± 6.27	<0.001
SLM	41.12 ± 7.65	50.48 ± 9.72	<0.001	39.88 ± 7.38	46.55 ± 8.15	<0.001
FFM	43.58 ± 8.05	53.44 ± 10.31	<0.001	42.26 ± 7.76	49.26 ± 8.59	<0.001
WHR	0.89 ± 0.04	0.96 ± 0.06	<0.001	0.90 ± 0.04	0.95 ± 0.05	<0.001
PBF	30.26 ± 6.65	37.07 ± 6.88	<0.001	31.17 ± 5.80	35.09 ± 6.52	<0.001
BMR	1311.22 ± 173.95	1524.39 ± 222.60	<0.001	1282.84 ± 167.69	1434.03 ± 185.50	<0.001
VFA	89.79 ± 24.79	149.98 ± 43.30	<0.001	93.48 ± 23.46	130.62 ± 36.54	<0.001
FFMI	16.25 ± 1.64	19.11 ± 2.09	<0.001	15.97 ± 1.57	18.29 ± 1.85	<0.001
FMI	7.07 ± 1.64	11.51 ± 3.29	<0.001	7.24 ± 1.42	10.04 ± 2.69	<0.001
SMI	6.65 ± 0.87	7.99 ± 1.01	<0.001	6.49 ± 0.86	7.57 ± 0.96	<0.001

BMI, body mass index; DM, diabetes mellitus; AST, aspartate; ALT, alanine aminotransferase; GGT, gamma-glutamyl transferase; CHO, cholesterol; HDL-c, high density lipoprotein cholesterol; LDL-c, low density lipoprotein cholesterol; BFM, body fat mass; SLM, soft lean mass; FFM, fat free mass; PBF, precent body fat; BMR, basal metabolic rate; WHR, waist-hip ratio; VFA, visceral fat area; BCM, body cell mass; BMC, bone mineral content; FFMI, fat free mass index; FMI, fat mass index; SMI, skeletal muscle index.

**Figure 2:**
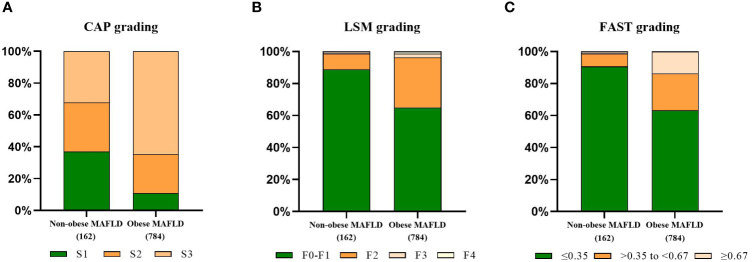
Comparison of fibrotic features of non-obese MAFLD and obese MAFLD. CAP-based steatosis grading **(A)**, LSM-based fibrosis grading **(B)**, proportion of non-alcoholic steatohepatitis judged by FAST score **(C)**.

To further clarify the changes in body composition in non-obese MAFLD patients, a comparison control group was established by nearest neighbor propensity score matching (1:2). After PSM, most baseline characteristics such as age and sex did not differ between the two groups, with 95 patients in the non-obese MAFLD group and 157 patients in the obese MAFLD group. Significant differences in body composition remained between the two groups (**Table 1**). Compared with obese MAFLD patients, non-obese MAFLD subjects had lower CAP value, LSM value, and FAST score (**Figure 2**). The percentages of S1-S3 estimated from the CAP measurements in the non-obese MAFLD group were as follows: S1, 37.04%; S2, 30.86%; and S3, 32.1% (**Figure 2A**). In the obese MAFLD group, the percentages were as follows: S1, 10.84%; S2, 24.74%; and S3, 64.41% (**Figure 2A**). The ratios of F0-F4 estimated from LSM measurements were: F0-F1, 88.89%; F2, 9.88%; and F3, 1.23% in the non-obese MAFLD group (**Figure 2B**). The ratios of F0-F4 in the obese MAFLD group were: F0-F1, 65.05%; F2, 31.25%; F3, 2.3%; and F4, 1.4% (**Figure 2B**). With FAST score ≥0.67 as the threshold, 13.65% of the obese MAFLD subjects could not exclude liver fibrosis, while only 1.23% of the non-obese group (**Figure 2C**).

2.Comparison of clinical characteristics and metabolic profiles between non-obese MAFLD patients and non-obese healthy controls

There were 263 subjects with BMI<25 kg/m^2^. They were divided into two groups: non-obese MAFLD and non-obese healthy controls. Non-obese MAFLD patients had higher age, BMI, ALT, GGT, UA, TG, LDL, CAP, FAST score, BFM, WHR, PBF, VFA, and FMI than non-obese healthy controls, but lower HDL using univariate analysis (**Table 2**). After PSM (1:2), there were no significant differences in age and gender between the two groups. There were 43 non-obese healthy controls and 95 non-obese MAFLD patients, but there were still significant differences in body composition such as BFM, WHR, PBF, VFA and FMI (**Table 2**). Using binary logistic regression analysis, higher GGT and lower HDL were associated with non-obese MAFLD (**Table 3**).

**Table 2:** Comparison of clinical characteristics and body composition between non-obese MAFLD and non-obese healthy controls.

Characteristic	Unmatching			Matching		
	Non-obese MAFLD (n = 162)	Non-obese healthy controls (n = 101)	*P* value	Non-obese MAFLD (n = 95)	Non-obese healthy controls (n = 43)	*P* value
Demography						
Age, year	50.45 ± 12.33	45.79 ± 13.88	0.005	58.81 ± 7.13	58.77 ± 5.48	0.972
Male, *n* (%)	70 (43.2%)	36 (35.6%)	0.224	35(36.84%)	15(34.88%)	0.825
Waist, cm	83.44 ± 4.91	79.34 ± 6.44	0.000	83.39 ± 4.95	79.04 ± 6.11	<0.001
BMI	23.32 ± 1.32	22.24 ± 2.14	<0.001	23.21 ± 1.22	22.37 ± 1.55	0.001
DM, *n* (%)	113 (69.8%)	72 (71.3%)	0.791	76(80.00%)	38(88.37%)	0.229
HTN, *n* (%)	40 (24.7%)	19 (18.8%)	0.266	33(34.74%)	14(32.56%)	0.802
Cardiovascular and cerebrovascular diseases, *n* (%)	5 (3.1%)	9 (8.9%)	0.041	5(5.26%)	7(16.28%)	0.033
Laboratory test						
Hb, g/L	141.49 ± 17.58	137.74 ± 13.86	0.070	139.62 ± 13.93	135.67 ± 12.39	0.113
PLT, 10^9/L	232.61 ± 66.93	219.81 ± 72.63	0.146	224.6 ± 61.50	199.23 ± 72.99	0.036
AST, U/L	23.79 ± 15.97	20.54 ± 8.51	0.060	22.17 ± 10.59	21.37 ± 6.61	0.650
ALT, U/L	28.82 ± 23.67	22.58 ± 14.94	0.019	24.68 ± 15.17	23.23 ± 11.85	0.580
ALP, U/L	83.98 ± 22.52	77.86 ± 38.85	0.107	85.00 ± 23.20	78.14 ± 18.87	0.091
GGT, U/L	41.60 ± 40.76	23.86 ± 19.68	<0.001	39.74 ± 42.49	23.86 ± 13.34	0.001
UA, μmol/L	323.78 ± 74.81	294.39 ± 86.27	0.004	313.45 ± 76.57	275.16 ± 76.74	0.007
Triglycerides, mmol/L	1.92 ± 1.46	1.32 ± 1.28	0.001	1.85 ± 1.22	1.21 ± 0.61	0.001
CHO, mmol/L	5.01 ± 1.14	4.81 ± 1.06	0.158	4.99 ± 1.23	4.77 ± 1.15	0.312
HDL-c, mmol/L	1.34 ± 0.31	1.50 ± 0.338	<0.001	1.37 ± 0.32	1.53 ± 0.28	0.003
LDL-c, mmol/L	2.88 ± 0.79	2.67 ± 0.77	0.042	2.86 ± 0.86	2.64 ± 0.89	0.161
Glucose, mmol/L	7.00 ± 2.40	6.43 ± 2.31	0.058	7.27 ± 2.39	6.58 ± 2.14	0.108
CAP, dB/m	280.30 ± 34.72	203.67 ± 25.88	<0.001	277.68 ± 33.43	203.72 ± 30.59	<0.001
LSM, kPa	5.39 ± 1.78	5.16 ± 1.92	0.321	5.37 ± 1.81	5.32 ± 1.62	0.862
FIB-4	1.08 ± 0.54	1.12 ± 0.74	0.595	1.29 ± 0.53	1.56 ± 0.72	0.595
FAST	0.13 ± 0.13	0.24 ± 0.43	0.002	0.11 ± 0.13	0.23 ± 0.26	<0.001
Body composition						
BFM	18.57 ± 3.59	16.23 ± 4.73	<0.001	18.82 ± 3.13	16.32 ± 3.82	<0.001
SLM	41.12 ± 7.65	40.57 ± 6.18	0.539	39.88 ± 7.38	39.49 ± 6.45	0.768
FFM	43.58 ± 8.05	43.03 ± 6.50	0.563	42.26 ± 7.76	41.88 ± 6.78	0.782
SMM	23.77 ± 4.89	23.33 ± 3.94	0.444	22.89 ± 4.71	22.59 ± 4.10	0.713
WHR	0.89 ± 0.04	0.86 ± 0.05	<0.001	0.90 ± 0.04	0.86 ± 0.04	<0.001
PBF, %	30.26 ± 6.65	27.26 ± 6.88	0.001	31.17 ± 5.80	28.13 ± 6.22	0.006
BMR	1311.22 ± 173.95	1299.37 ± 140.19	0.564	1282.84 ± 167.69	1274.6 ± 146.142	0.782
VFA, cm^2^	89.79 ± 24.79	75.96 ± 26.64	<0.001	93.48 ± 23.46	77.62 ± 22.29	<0.001
FFMI	16.25 ± 1.64	16.11 ± 1.59	0.495	15.97 ± 1.57	16.06 ± 1.49	0.773
FMI	7.07 ± 1.64	6.14 ± 1.84	<0.001	7.24 ± 1.42	6.32 ± 1.55	0.001
SMI	6.65 ± 0.87	6.59 ± 0.76	0.576	6.49 ± 0.86	6.51 ± 0.78	0.903

BMI, body mass index; DM, diabetes mellitus; AST, aspartate; ALT, alanine aminotransferase; GGT, gamma-glutamyl transferase; CHO, cholesterol; HDL-c, high density lipoprotein cholesterol; LDL-c, low density lipoprotein cholesterol; BFM, body fat mass; SLM, soft lean mass; FFM, fat free mass; PBF, precent body fat; BMR, basal metabolic rate; WHR, waist-hip ratio; VFA, visceral fat area; BCM, body cell mass; BMC, bone mineral content; FFMI, fat free mass index; FMI, fat mass index; SMI, skeletal muscle index.

**Table 3:** Factors associated with non-obese MAFLD categorized using binary logistic regression.

Variables compared with non-obese healthy control	AOR	95% CI	*P* value
Cardiovascular and cerebrovascular diseases	0.482	0.127-1.823	0.282
ALT, U/L	1.000	0.984-1.017	0.984
GGT, U/L	0.975	0.959-0.990	0.002
UA, μmol/L	0.997	0.994-1.001	0.178
TG, mmol/L	0.911	0.704-1.180	0.481
HDL-c, mmol/L	4.931	1.697-14.328	0.003
LDL-c, mmol/L	0.686	0.469-1.003	0.052
BFM	1.129	0.838-1.520	0.425
VFA, cm^2^	0.981	0.942-1.022	0.350
PBF	1.163	0.947-1.428	0.149
FMI	0.404	0.141-1.161	0.092

ALT, alanine aminotransferase; GGT, gamma-glutamyl transferase; HDL-c, high density lipoprotein cholesterol; LDL-c, low density lipoprotein cholesterol. BFM, body fat mass; VFA, visceral fat area; FMI, fat mass index.

3.Comparison of correlation index of islet function between non-obese MAFLD patients and non-obese healthy controls.

In this study population, there were 182 non-obese subjects with available data on the correlation index of the islet function. Of them, 109 patients were diagnosed with non-obese MAFLD. Non-obese MAFLD patients had higher age, ALT, GGT, and fasting C-peptide than non-obese healthy controls (**Table 4**). A stratified analysis excluding the influence of age showed that FCP was still different between the two groups (**Supplementary Table 1**).

**Table 4:** Comparison of correlation index of islet function between non-obese MAFLD and non-obese healthy controls.

	Non-obese MAFLD (n=109)	Non-obese healthy control (n=73)	*P* value
Age, year	53.11 ± 11.48	47.64 ± 12.93	0.004
Male, *n* (%)	52 (47.7%)	29 (39.7%)	0.288
AST, U/L	23.04 ± 16.93	19.90 ± 7.53	0.092
ALT, U/L	28.42 ± 26.55	22.29 ± 13.64	0.043
GGT, U/L	38.19 ± 38.96	22.85 ± 17.85	<0.001
Glucose, mmol/L	7.39 ± 2.45	6.75 ± 2.53	0.095
HbA1c, %	7.44 ± 1.89	7.04 ± 2.07	0.191
FCP, ng/ml	1.63 ± 7.99	1.39 ± 0.68	0.028
Fasting insulin, uIU/ml	8.22 ± 5.70	7.88 ± 13.31	0.837
HOMA-IR	2.60 ± 1.89	2.21 ± 3.52	0.387

AST, aspartate; ALT, alanine aminotransferase; GGT, gamma-glutamyl transferase; FCP, Fasting C-peptide.

## Discussion

In our study population, the prevalence of non-obese MAFLD was 13.90% among MAFLD patients. Studies on the incidence of non-obese MAFLD were heterogeneous. A meta-analysis of 10576383 people showed that 12.1% of the general population had non-obese NAFLD (12). A study in Asian populations showed that 21.6% of NAFLD patients were non-obese (13). We found that non-obese MAFLD was older than obese patients. Interestingly, although patients with non-obese MAFLD have lower waist circumference and BMI than those with obese MAFLD, they have a higher incidence of diabetes. A meta-analysis of 15 studies showed that lean and obese patients with NAFLD share a common altered metabolic and cardiovascular profile (14). Although past studies have shown that non-obese NAFLD occurs more in females (15), no significant gender differences were found in our study. A previous meta-analysis also confirmed this view (16).

In general, the metabolic abnormalities of non-obese MAFLD, although milder than those of obese MAFLD, are more pronounced than in non-obese healthy people. Previous studies have shown that the occurrence of NAFLD is strongly associated with metabolic dysfunction, but the number of metabolically unhealthy patients with non-obese NAFLD is significantly less than that of obese NAFLD patients (17). Our study showed that GGT and HDL-c were significantly associated with non-obese MAFLD subjects when compared with non-obese healthy controls. A previous study found a nonlinear association of the GGT/HDL-c ratio with NAFLD (18). Therefore, GGT and HDL-c may be predictors of non-obese MAFLD. However, our study showed that non-obese MAFLD had higher Fasting C-peptide than non-obese healthy controls. Studies have shown that fasting C-peptide is an independent predictor of NAFLD (19). We confirmed that the incidence of Cardiovascular and cerebrovascular diseases between non-obese MAFLD and obese MAFLD is comparable. A cross sectional study showed that non-obese NAFLD had no better cardio-metabolic risk profile than obese NAFLD in patients with T2DM (20).

Data on histological severity are controversial, but they can develop the full spectrum of liver disease associated with nonalcoholic steatohepatitis NASH (21). The risk of developing liver fibrosis in patients with MAFLD increases with body weight. Our study confirmed that LSM levels in non-obese MAFLD were between obese MAFLD and non-obese healthy controls. Obesity increases the risk of liver fibrosis in patients with MAFLD. The development of liver fibrosis in patients with non-obese MAFLD may be associated with changes in the intestinal flora. Studies have shown that *Ruminococcaceae* and *Veillonellaceae* are the major microbiota associated with fibrosis severity in non-obese subjects (22).

In this study, we found that non-obese individuals with MAFLD had higher VFA and PBF than healthy individuals, suggesting that fat accumulation plays a key role in the development of MAFLD in non-obese individuals. This finding is consistent with the results of previous studies (23). Studies have shown that the increase in visceral fat area is closely related to the occurrence of hepatic insulin resistance and NASH, and the change in visceral fat is an early predictor of the progression of MAFLD (24). A meta-analysis indicated that the SMI level in patients with NAFLD was lower than in healthy people, and sarcopenia is associated with NAFLD (25). However, there was no significant difference in SMI between non-obese MAFLD subjects and healthy controls in our study, which may be related to the small sample size.

The pathogenesis and pathophysiology of non-obese MAFLD are still unclear. Studies have shown that the occurrence of non-obese MAFLD is related to genetic susceptibility, intestinal flora, impaired glucose tolerance, fructose intake (26), and environmental factors (27). Insulin resistance is generally thought to be an important mechanism in the pathogenesis of MAFLD (28). The progression of lean NAFLD is affected by multiple epigenetic mechanisms. A large number of studies have shown that carrying the PNPLA3 rs738409 gene is strongly associated with the occurrence of lean NAFLD (29). Changes in the gut microbiome may be another risk factor for the development of lean MAFLD.

There are currently no specific drugs for the treatment of non-obese MAFLD, and weight loss and lifestyle management remain the first choices. A 5% weight loss significantly improved hepatic steatosis in non-obese subjects (30). Lifestyle intervention is effective in the treatment of non-obese MAFLD. Previous research showed that remission of NAFLD can be achieved in 67% of non-obese patients after lifestyle intervention (31). In lean patients with NAFLD, lifestyle intervention, including exercise, diet modification, and avoidance of fructose- and sugar-sweetened drinks, to target a modest weight loss of 3%-5% is suggested (32). Liraglutide has a certain effect on lean NASH, but the benefits of lean NAFLD need further research (33). The therapeutic role of glucagon-like peptide-1 agonists and sodium-glucose cotransporter-2 inhibitors in the management of lean NAFLD is not fully defined and requires further investigation.

Our study had some strengths. First, this was a study to assess the association among metabolic factors, body composition, and non-obese MAFLD. Second, our study population included MAFLD patients and healthy controls. However, some limitations should also be addressed. First, the diagnostic accuracy of Fibroscan decreased in people with a BMI >30 kg/m^2^. Second, this was a cross-sectional study and was unable to demonstrate the causal relationship.

In summary, the prevalence of non-obese MAFLD is 13.90% and is more likely to occur in the elderly. Non-obese MAFLD patients had different metabolic profiles and body composition compared with obese MAFLD subjects. Furthermore, HDL-c and GGT were associated with non-obese MAFLD patients, suggesting HDL-c and GGT might be predictors of disease progression in MAFLD patients. In addition, weight loss may be a key link in preventing fatty liver from progressing to liver fibrosis.

## Data availability statement

The raw data supporting the conclusions of this article will be made available by the authors, without undue reservation.

## Ethics statement

The studies involving humans were approved by The Ethics Committee of Jiangsu Provincial Hospital of Traditional Chinese Medicine. The studies were conducted in accordance with the local legislation and institutional requirements. The participants provided their written informed consent to participate in this study.

## Author contributions

YZ: Data curation, Writing – original draft, Formal analysis, Writing – review & editing. LX: Data curation, Writing – original draft. FQ: Data curation, Writing – original draft. YC: Writing – original draft, Formal analysis. WZ: Writing – original draft, Formal Analysis. TL: Writing – original draft, Formal analysis. XZ: Writing – review & editing, Writing – original draft.
