# Supplementary figures and images for: The metabolic profiles and body composition of non-obese metabolic associated fatty liver disease
Source: Front Endocrinol (Lausanne). 2024 Feb 5;15:1322563. doi: 10.3389/fendo.2024.1322563 (PMC10876088; doi:10.3389/fendo.2024.1322563)

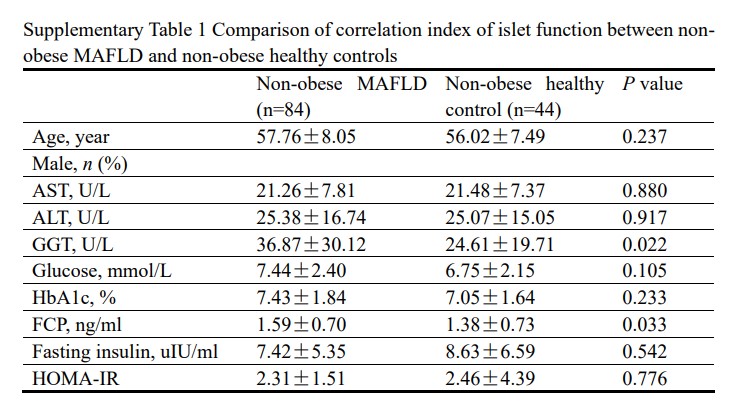

Supplement: Supplementary file 1 [file Image_1.jpeg]
